# Supplementary material for: Vitamin D Modified DSS-Induced Colitis in Mice via STING Signaling Pathway
Source: Biology (Basel). 2025 Jun 18;14(6):715. doi: 10.3390/biology14060715 (PMC12190092; doi:10.3390/biology14060715)
Supplement: Supplementary file 1 [file biology-14-00715-s001.zip › Supplementary Materials S1: 16s-report of gut microbiota/Groups/diff_analysis/TukeyHSD/Tukey_METACYC.pdf]

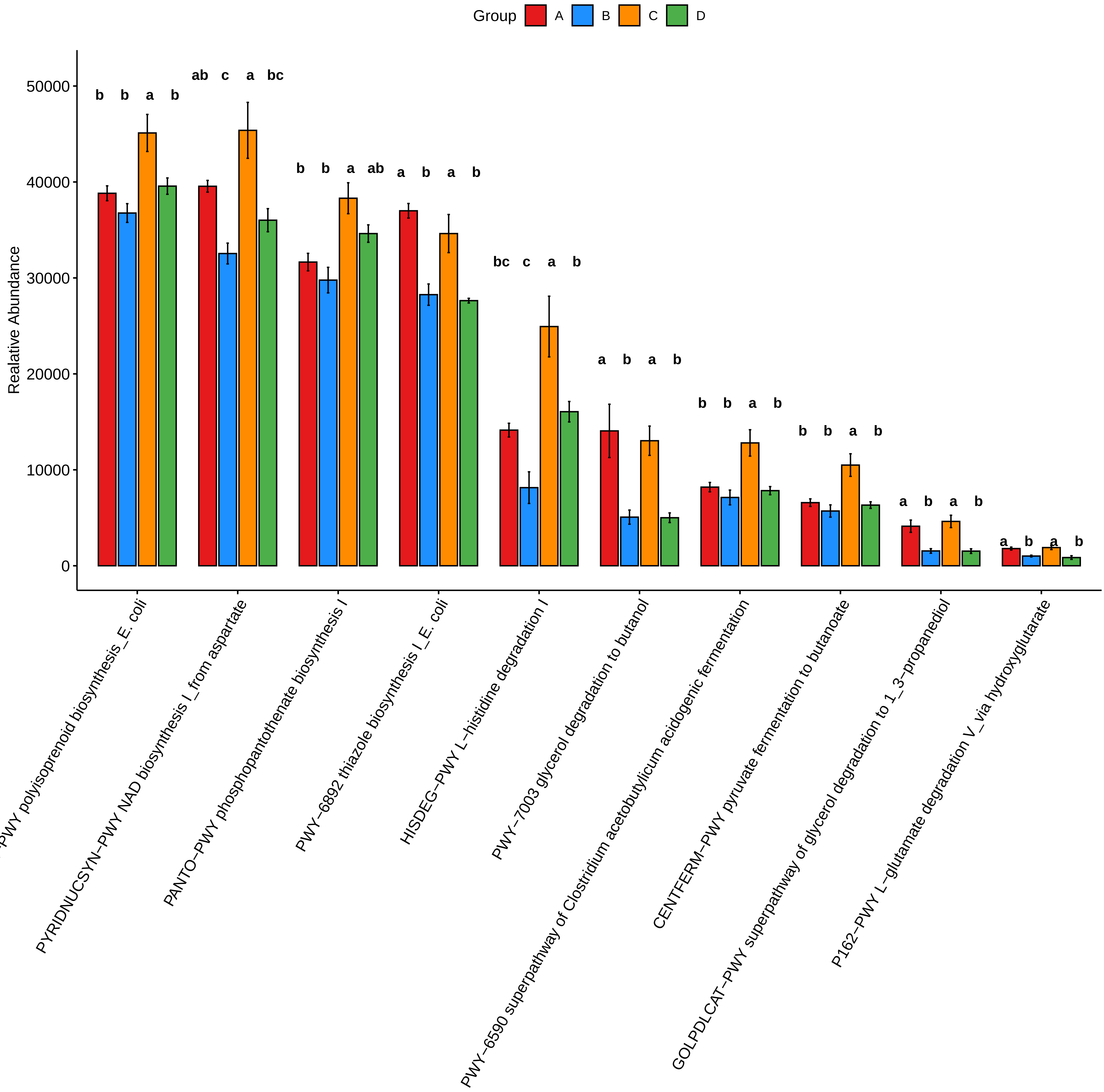
Group
A
B
C
D
Realative Abundance
0
10000
20000
30000
40000
50000
b b a b
ab c a bc
b b a ab
a b a b
bc c a b
a b a b
b b a b
b b a b
a b a b
a b a b
PWY polyisoprenoid biosynthesis_E. coli
PYRIDNUCSYN−PWY NAD biosynthesis I_from aspartate
PANTO−PWY phosphopantothenate biosynthesis I
PWY−6892 thiazole biosynthesis I_E. coli
HISDEG−PWY L−histidine degradation I
PWY−7003 glycerol degradation to butanol
PWY−6590 superpathway of Clostridium acetobutylicum acidogenic fermentation
CENTFERM−PWY pyruvate fermentation to butanoate
GOLPDLCAT−PWY superpathway of glycerol degradation to 1_3−propanediol
P162−PWY L−glutamate degradation V_via hydroxyglutarate
